# Supplementary material for: Outcomes of open versus single-incision laparoscopic totally extraperitoneal inguinal hernia repair using propensity score matching: A single institution experience
Source: PLoS One. 2021 Jan 28;16(1):e0246189. doi: 10.1371/journal.pone.0246189 (PMC7842936; doi:10.1371/journal.pone.0246189)
Supplement: Attachment — Submitted filename: Response to Reviewers.docx [file pone.0246189.s002.docx]

**Author’s response to Reviewer**

First of all, we greatly appreciate your favorable review of our manuscript. In addition, we would also like to thank you for your valuable comments and suggestions. In response to the points that you raised, we would like to offer the following answers.

Journal Requirements:

When submitting your revision, we need you to address these additional requirements.

1. Please ensure that your manuscript meets PLOS ONE's style requirements, including those for file naming. The PLOS ONE style templates can be found at https://journals.plos.org/plosone/s/file?id=wjVg/PLOSOne_formatting_sample_main_body.pdf and

https://journals.plos.org/plosone/s/file?id=ba62/PLOSOne_formatting_sample_title_authors_affiliations.pdf

Response: The manuscript was revised according to the journal’s style requirements.

2. Please provide additional details regarding participant consent. In the ethics statement in the Methods and online submission information, please ensure that you have specified (1) whether consent was informed and (2) what type you obtained (for instance, written or verbal, and if verbal, how it was documented and witnessed). If your study included minors, state whether you obtained consent from parents or guardians. If the need for consent was waived by the ethics committee, please include this information. If you are reporting a retrospective study of medical records or archived samples, please ensure that you have discussed whether all data were fully anonymized before you accessed them and/or whether the IRB or ethics committee waived the requirement for informed consent. If patients provided informed written consent to have data from their medical records used in research, please include this information.

Response: The following details were added regarding participant consent on the Materials and Methods section:

- Medical records were retrospectively reviewed to identify patients who underwent surgery for inguinal hernia at National Health Insurance Service Ilsan Hospital between November 2016 and November 2018. Exclusion criteria were as follows: (1) under 18 years of age, (2) underwent CTEP repair, (3) emergency operation for treatment of acute bowel incarceration, (4) femoral hernia, and (5) combined operation with inguinal hernia repair. The current study was performed after approval by the Institutional Review Boards of National Health Insurance Service Ilsan Hospital (NHIMC2019-03-004). This was a retrospective observational study; therefore, the requirement for informed consent was waived by the Institutional Review Boards. The raw and analysis-ready datasets were anonymized by removing all personally identifiable information.

3. Thank you for stating the following in the Funding Section of your manuscript:

"This study was supported by a faculty research grant from the National Health Insurance Service Ilsan Hospital (NHIMC 2019CR027)." We note that you have provided funding information that is not currently declared in your Funding Statement. However, funding information should not appear in the Acknowledgments section or other areas of your manuscript. We will only publish funding information present in the Funding Statement section of the online submission form.

Please remove any funding-related text from the manuscript and let us know how you would like to update your Funding Statement. Currently, your Funding Statement reads as follows:

"The funders had no role in study design, data collection and analysis, decision to publish, or preparation of the manuscript." Please include your amended statements within your cover letter; we will change the online submission form on your behalf.

Response: A statement related to the funding is removed from the title page. Please keep the current funding statement. "The funders had no role in study design, data collection and analysis, decision to publish, or preparation of the manuscript." We added the funding statements in the cover letter.

4. Your ethics statement should only appear in the Methods section of your manuscript. If your ethics statement is written in any section besides the Methods, please delete it from any other section.

Response: We removed the ethic statement in any section besides the methods in the revised manuscript.

**Review Comments to the Author**

**Reviewer #1:** It is a well written manuscript about an important new surgical technique of repair of inguinal hernia. The language is well written in standard format On first impression I would have believed that there is no need to compare single-incision technique with open repair rather than comparing with conventional laparoscopic hernia repairs, when already similar comparisons have been published with better results for laparoscopic procedures. but I would agree with the authors that there should be independent published data about comparing the single-incision technique with open repair. The data analysis is well done. Over all its a very well presented manuscript.

Response: Thank you very much for the comments.

**Reviewer #2:** The article is a good read, clear, concise with good written English.

Ideally, evidence-based medicine should use prospective, randomized studies. These studies are difficult and expensive and are therefore rarely available and we resort to modified observational studies such as propensity score matching.
The most important limitation of propensity score methods is that although they can bring an equilibrium to the observed baseline covariates between the two groups mentioned in the study, they do nothing to evaluate and balance the unmeasured characteristics and confounders. Taking these aspects into consideration, unlike randomized control trials, propensity score analyses are limited in interpretation exactly due to these unmeasured confounding variables. This can lead to biased results and caution needs to be taken when the results are interpreted. The authors should mention these aspects in the study limitations.

Regarding the title, the authors should mention that this is a single institutional study.
The authors mention only 1 intraoperative incident in the SILTEP group, the bladder lesion. In three years of observation there were no conversions to TAP due to peritoneum rupture or conversion to open surgery?

Also the authors need to make available the data used for analysis of course by maintaining patient anonymity as to comply with the Plos One guidelines and to ensure the reproductivity of the results.

Response:

- The following details on the limitations of propensity score matching was added on the discussion section:

The present study does have several limitations. First, we did not show data on cosmetic results, quality of life, and long-term outcomes because of a short follow-up period. Second, the study was designed retrospectively and various analyses for postoperative pain were not possible. Third, although propensity score matching reduced potential selection bias, the possibility of remaining unmeasured confounding variables may still be present. Thus, this may lead to biased results, and caution needs to be taken while interpreting these results. Finally, we did not analyze and compare the costs of SILTEP and open repair.

- The title has been revised as requested as follows:
Outcomes of open versus single-incision laparoscopic totally extraperitoneal inguinal hernia repair using propensity score matching : a single institution experience

- There was only 1 patient with open conversion due to peritoneal tearing. Surgery with a single port is usually performed even if peritoneal tearing occurs during the operation. Peritoneal tears during surgery can significantly impair the progress of surgery, and the operation time is increased. However, when peritoneal tearing occurred, we performed peritoneal repair using intracorporeal laparoscopic suture, endoloop, and a hemolok clip. Thus, it was possible to continue the operation with a single port without open conversion or conversion to TAP.

- Data set was added to the revised manuscript on the supporting information section.
